# A data science roadmap for open science organizations engaged in early-stage drug discovery

**DOI:** 10.1038/s41467-024-49777-x

**Published:** 2024-07-05

**Authors:** Kristina Edfeldt, Aled M. Edwards, Ola Engkvist, Judith Günther, Matthew Hartley, David G. Hulcoop, Andrew R. Leach, Brian D. Marsden, Amelie Menge, Leonie Misquitta, Susanne Müller, Dafydd R. Owen, Kristof T. Schütt, Nicholas Skelton, Andreas Steffen, Alexander Tropsha, Erik Vernet, Yanli Wang, James Wellnitz, Timothy M. Willson, Djork-Arné Clevert, Benjamin Haibe-Kains, Lovisa Holmberg Schiavone, Matthieu Schapira

**Affiliations:** 1 Structural Genomics Consortium, Department of Medicine, Karolinska University Hospital and Karolinska Institutet, Stockholm, Sweden; 2 Structural Genomics Consortium, University of Toronto, Toronto, ON Canada; 3 Discovery Sciences, R&D, AstraZeneca, Gothenburg, Sweden & Department of Computer Science and Engineering, Chalmers University of Technology, Gothenburg, Sweden; 4 Bayer AG Research and Development, Computational Molecular Design, Berlin, Germany; 5 European Molecular Biology Laboratory, European Bioinformatics Institute (EMBL-EBI), Wellcome Genome Campus, Hinxton, UK; 6 Open Targets, Wellcome Genome Campus, Hinxton, Cambridgeshire UK; 7 European Bioinformatics Institute (EMBL-EBI), Wellcome Genome Campus, Hinxton, Cambridge UK; 8 Centre for Medicines Discovery, NDM, University of Oxford, Oxford, UK; 9 Institute of Pharmaceutical Chemistry, Johann Wolfgang Goethe University, Frankfurt am Main, 60438, Germany & Structural Genomics Consortium (SGC), Buchmann Institute for Life Sciences, Johann Wolfgang Goethe University, Frankfurt am Main, Germany; 10 National Library of Medicine, National Institutes of Health, Bethesda, MD USA; 11 Pfizer Worldwide Research, Development & Medical, Cambridge, MA USA; 12 Pfizer, Worldwide Research, Development and Medical, Machine Learning & Computational Sciences, Berlin, Germany; 13 Department of Discovery Chemistry, Genentech, Inc., South San Francisco, CA USA; 14 Laboratory for Molecular Modeling, Division of Chemical Biology and Medicinal Chemistry, UNC Eshelman School of Pharmacy, University of North Carolina, Chapel Hill, North Carolina USA; 15 Digital Science & Innovation, Novo Nordisk A/S, Maaloev, Denmark; 16 Structural Genomics Consortium, UNC Eshelman School of Pharmacy, University of North Carolina at Chapel Hill, Chapel Hill, NC USA; 17 Princess Margaret Cancer Centre, University Health Network, Toronto, ON Canada; 18 Department of Medical Biophysics, University of Toronto, Toronto, ON Canada; 19 Vector Institute for Artificial Intelligence, Toronto, ON Canada; 20 Discovery Biology, Discovery Sciences, R&D, AstraZeneca, Gothenburg, Sweden; 21 Department of Pharmacology & Toxicology, University of Toronto, Toronto, ON Canada

**Keywords:** Research data, Drug discovery and development

## Abstract

The Structural Genomics Consortium is an international open science research organization with a focus on accelerating early-stage drug discovery, namely hit discovery and optimization. We, as many others, believe that artificial intelligence (AI) is poised to be a main accelerator in the field. The question is then how to best benefit from recent advances in AI and how to generate, format and disseminate data to enable future breakthroughs in AI-guided drug discovery. We present here the recommendations of a working group composed of experts from both the public and private sectors. Robust data management requires precise ontologies and standardized vocabulary while a centralized database architecture across laboratories facilitates data integration into high-value datasets. Lab automation and opening electronic lab notebooks to data mining push the boundaries of data sharing and data modeling. Important considerations for building robust machine-learning models include transparent and reproducible data processing, choosing the most relevant data representation, defining the right training and test sets, and estimating prediction uncertainty. Beyond data-sharing, cloud-based computing can be harnessed to build and disseminate machine-learning models. Important vectors of acceleration for hit and chemical probe discovery will be (1) the real-time integration of experimental data generation and modeling workflows within design-make-test-analyze (DMTA) cycles openly, and at scale and (2) the adoption of a mindset where data scientists and experimentalists work as a unified team, and where data science is incorporated into the experimental design.

## Abstract (web summary)

Artificial intelligence is greatly accelerating research in drug discovery, but its development is still hindered by the lack of available data. Here the authors present data management and data science recommendations to help reach AI’s potential in the field.

## Introduction

A growing federation of scientists share the goal of generating chemical modulators for all druggable human proteins by the year 2035^1^. Under the umbrella name Target 2035, the rationale for this initiative is that selective chemical probes are precious tools to study functional genomics and reveal novel targets for unmet medical needs. Artificial intelligence (AI) may be the accelerator needed to reach this over-ambitious goal. However, AI can only fulfil this promise if trained on datasets that are large, reliable and machine interpretable^2^. The Structural Genomics Consortium (SGC) is a multi-national open science public-private partnership invested in the goal of Target 2035. In January-February 2023, a working group of experimentalists, data management experts and data scientists from the public and private sectors discussed the best mechanisms to enable data science for hit discovery and optimization. The resulting document provides a set of best practices for data management, data dissemination and data science in early, pre-competitive drug discovery, and a template for efforts in the Target 2035 initiative. Above all, we believe that a novel mindset is necessary where data science is fully integrated within experimental plans and workflows, even before experiments are conducted. The field expertise resides with experimentalists, while data scientists develop the knowledge discovery engine. Both must speak the same language and be engaged at each step of data generation, curation, modelling, and data- and model-driven experimental design. This white paper provides strategic and operational guidance for the SGC and Target 2035 to fuel and benefit from progress in the data science of drug discovery. We hope it will inspire further discussion in other research institutions ready to contribute open science datasets and embrace AI for early-stage drug discovery.

## Best practices and recommendations for data management

### Planning early for data science applications

It is universally recognized that all data generation/collection, at least from the public sector, needs to adhere to FAIR principles: Findability, Accessibility, Interoperability and Reusability^3^. The next frontier is to generate, process and curate data explicitly with a focus on computational knowledge discovery. To achieve this goal, it will be essential to work across boundaries and have data scientists and experimentalists come together and agree on how to structure, curate and annotate data to enable data mining.

### Defining precise ontologies and vocabulary

To be machine-interpretable, data should be ontologized (Nicola Guarino 1998^4^), which means that the different categories and relationships of data should be well-defined (Box 1). In other words, the data should be described with a pre-defined, formalized vocabulary to enable interpretation by machine learning models (e.g., for protein production and chemical screening), as discussed in more detail below. Descriptions should reflect datas quality, precision and/or reproducibility. “Negative” data as defined by experimentalists, meaning data that behave similarly to the negative control, should be collected with the same care as “positive” data and included in the experimental datasets. For some data ontologies it is inherent to the process to collect the “negative” data, for example in compound screening, whereas failed protein expression is rarely captured, leading to missed opportunities in data science (and future experiments). Metrics such as the number of experimental repeats should be provided to assess the risk of false positive and negative data points. A concrete example is given in the DNA Encoded Libraries (DEL) section below.

Box 1 keywords and concepts that are described in the text

**Table:** 

Keyword/abbreviation	Description
API—application programming interfaces	Software interface between two different software systems. For example, data in a LIMS system can be linked to a protocol in an ELN via the API.
Controlled vocabulary	Set of formalized terms used to describe data types. Often accessed via drop-down menus in a table format. This enables ML-ready data.
Data curation	Data curation is the organisation, annotation and standardisation of different datasets that reside within the same data ontology (e.g.: protein production datasets).
Database schema	A blueprint of the database architecture which commonly includes a visual representation. The schema does not contain any data, instead, it defines how data are organised. Most databases contain tables, and the database schema describes the relationships between the different tables.
ELN—electronic lab notebook	An electronic notebook is used in the laboratory to record material and methods, results, and summary. Depending on the field, ELNs include varying levels of free-text annotations. For example, synthetic reactions recorded in ELNs can have a structured format and be machine-readable, while comments or attachments on protein production would be less machine-readable than a LIMS system.
LIMS—laboratory information management system	A LIMS is a software to capture data related to experimental workflows. For example, in a protein production workflow, data related to the cloning of a gene, protein expression, purification, and quality control is captured. Data is generally entered in a controlled vocabulary format allowing the data to be easily searchable. LIMS systems can be integrated with laboratory automation allowing automatic upload of data into the system.
Metadata	Metadata adds information/describes the data generated. In an ELN or LIMS system metadata include when an experiment was performed and by whom.
Multimodal data	Data of different types are typically derived using different experimental approaches to investigate a common biological system or sample.
Ontology	Ontology within data science aims to describe the connectivity of different methods and data in a machine-readable format. For example, Bioassay Ontology (BAO) describes biological screening assays and their results, including high-throughput screening data, for the purpose of categorizing assays and associated data https://www.ebi.ac.uk/ols/ontologies.
Raw data	Raw data is unprocessed data. Examples include direct reads from instruments in the lab.
Refined data	Refined data has been processed and/or interpreted
Relational database	It is a database where data is stored in one or more tables linked to one another.

### Aiming for a centralized database architecture

As much as possible, a unified data structure should be adopted across all activities within data-generating institutions, such as the SGC. Database schemas for protein production and library screening, as two examples, should be shared across laboratories. For the SGC, this will facilitate the generation of global datasets with increased impact. Adoption of a shared data management tool will help achieve this goal and should be encouraged. One potential benefit of establishing a unified, robust and highly populated database architecture might be that other organizations generating similar data adopt this schema and contribute to an expanding dataset. Importantly, the data schema should be compatible with that of well-established repositories such as ChEMBL^5^ to facilitate data dissemination.

### ELN and LIMS, data management cornerstones for experimentalists

Many organisations use both a laboratory information management system (LIMS) and an electronic lab notebook (ELN) for the management of data and protocols. In the former, the data from a specific workflow are captured in a controlled vocabulary format, whereas in the latter the actual experiment is generally described in free-text form, including methods and results. To standardise ELN write-ups, the use of pre-defined templates is recommended. For the experimentalist, the first step in the data management process is to design and record the experimental setup, materials and methods, results and summary in a “write-up” in the ELN. Sub-division of the ELN into project-specific notebooks should be considered, as it facilitates access and enables team collaboration within a project, across geographical and/or organisational boundaries. The write-up in the ELN is followed by the registration of the raw and refined data into the LIMS system. This can be done manually by the scientist, or by automated data upload from the instrument to the LIMS system. Preferably, the ELN should be integrated with the LIMS system via an application programming interface (API) to link data to protocols. Deployment of data management systems as described above enables stakeholders in organisations to quickly find the information/data linked to a specific project.

### Pushing the boundaries of data recording with lab automation

In principle, even highly detailed metadata such as purity of reagents or ambient temperature for chemical reactions, to name just a few, should be judiciously recorded. This level of compliance in experimental recording may eventually only be achieved with highly automated instrumentation, such as self-driving laboratories and/or SMART labs^6^. One can think and should prepare for a future where AI-operated laboratories generate large, rich and reliable training sets to build AI models that guide experimental design.

### Opening ELNs to data mining

Experimental protocols and conditions recorded within ELNs represent a rich source of information that should be open to data scientists. To achieve this goal, a concerted effort to make ELN records machine-readable should be considered. For instance, drop-down menus could be used to select catalysts in chemical reactions or reaction conditions. ELNs could also be a vehicle to flag failing chemical reactions, a critical knowledge that is otherwise never shared. As mentioned above, formatting records in ELNs to support data mining may often prove too demanding for experimentalists. One may envision a future where large language models are used instead to mine free text in ELNs to yield structured data. Here again, the adoption of a unified ELN and database system across geographical sites, or at least across sites focused on the same scientific activity (e.g., protein production or chemistry) is highly recommended.

### Deploying integrated ELN and LIMS solutions

Adopting integrated LIMS and ELN solutions from the same software provider is recommended. Examples include ICM Scarab, collaboratively developed by the SGC and MolSoft LLC, or CDD Vault offered by Collaborative Drug Discovery^7^. An established platform to register protocols is https://www.protocols.io/. Alternatively, databases from different vendors can be integrated via API. For example, the International Bioscience Information System (IBIS) for the definition of protocols, test parameters and target information with test results being reported and analysed in Genedata Screener^8^.

### Curation of legacy datasets

Integration of legacy data can be a powerful method to increase the size and coverage of available datasets. For example, the Stockholm and Toronto sites of the SGC manage large and complementary protein production datasets. Such legacy data need to be curated before being considered for any type of downstream data mining effort. A prerequisite is that data have been collected in a LIMS system using controlled vocabularies. The quality, ontologies and vocabulary of each dataset should be evaluated during the data curation effort^9^.

## Data management case studies

Experimentalists and data scientists should first agree on the data and meta-data to be recorded in the database. Indeed, data management operations, while guided by common principles, can vary significantly, depending on the experiment. Here, we detail specific data management operations for a low-to-medium throughput and a high throughput workflow: protein production and DNA Encoded Libraries (DEL) screening.

### Protein production

**Fig. 1:**
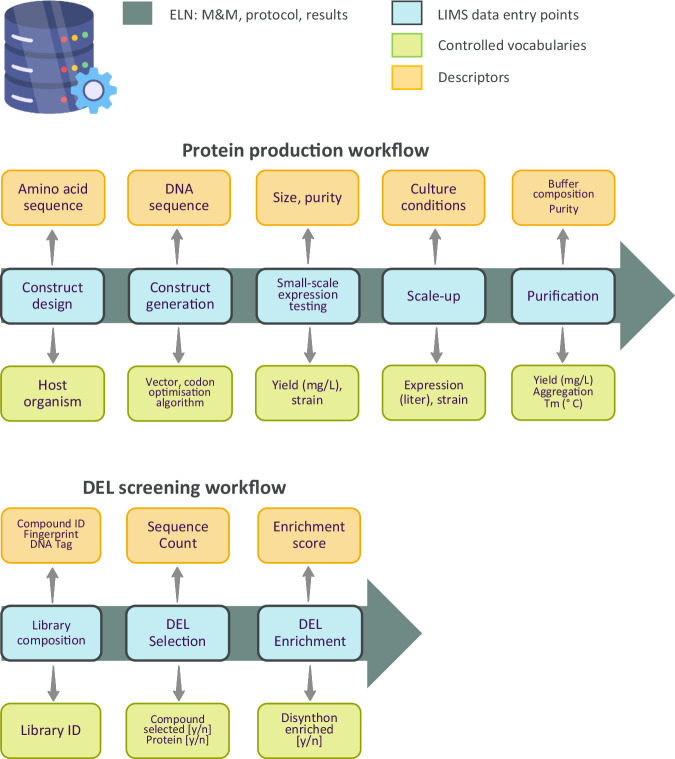
Data management workflows for protein production and chemical library screening. Controlled vocabulary and descriptors are used in data management workflows for protein production (top) and DEL screening (bottom). Materials & methods (M&M), protocols and results are recorded in an electronic lab notebook (ELN). DEL: DNA encoded library; ID: identifier; LIMS: laboratory information management system; Tm: melting temperature.

Protein production involves several steps including construct design, screening of experimental conditions at small-scale, large-scale expression, purification and quality control (QC). The associated data management workflow is illustrated in Fig. 1. First, constructs are designed for expression in host organisms such as *E. coli*, insect cells and mammalian cells. Since DNA sequences can be codon optimised for the host organism used for expression, it is critical to record the final DNA sequence of the expressed construct in the LIMS system. Constructs are often sourced from an external vendor with a success rate for cloning being almost 100%. Thus, there is no need to record the cloning success rate in the database. The next step is the small-scale expression and one-step affinity purification of the constructs in the selected host organism. At this step soluble expression yield should be captured in the LIMS system by controlled vocabulary, together with host strain, volume of culture and expression medium used. The registered data should be accompanied by a protein size descriptor. Protein yield and size are both typically assessed by performing denaturing gel electrophoresis, where yield and size compared to a protein standard are visualised. Yield can be estimated by quantifying the amount of the target protein compared to the protein standard in the stained gel, using densitometric analysis. At this stage, it is important to also capture the constructs that do not express soluble protein variants.

For a typical campaign, up to 96 different variants of a single protein are assessed to identify domain boundaries and tags that enable soluble protein expression. Commonly, up to 12 of the best-expressing samples might progress to larger-scale expression and purification. Again, experimental parameters (culture volume and yield) should be captured in the LIMS system, as often yields do not scale proportionately. The type of data will define whether they are entered via controlled vocabulary or descriptors. Data types include the aggregation status of purified protein samples measured by size exclusion chromatography (vocabulary: aggregated/non-aggregated), melting temperature, a surrogate for protein stability (descriptor: Tm in Celsius) and whether the protein identity was confirmed by mass spectrometry (vocabulary: yes/no). Buffer composition varies extensively and thus should be entered as free text/descriptor like the purity of the protein, which is an estimate by eye. Adherence to this data registration workflow will enable machine learning models to learn how the DNA sequence (and amino acid sequence) of protein construct affects the yield of protein expression, protein thermal stability and aggregation status.

Organizations such as the SGC could encourage partnering centres to implement similar data management protocols, leading to the generation of larger and more impactful collective datasets to empower machine learning. This may enable the development of powerful predictive models for construct design.

### DNA Encoded Library screening

Capitalizing on the success and efficiency of modern chemistry^10^ and next-generation sequencing^11^, DEL technology (conceptualized by Benner and Lerner more than 30 years ago^12^) is a useful screening technique for the identification of high-affinity ligands. DEL selection allows simultaneous screening of billions of compounds in small volumes, against a target in a single experiment. This allows for DEL selection to have a dramatically lower screening cost and shorter experimental runtime, with only minor costs coming from the sequencing of DNA from the screening results^13–15^. The efficiency of DEL screening in comparison to that of the conventional HTS technologies has been extensively discussed and reviewed^14,16,17^.

This substantial increase in time and cost efficiency comes with some trade-offs. Notably, DEL screening only detects the binding of a compound to a target and neither accurately determines association constants nor detects whether such binding will cause the desired biological effect. In addition, the small concentrations used and reliance on PCR amplification of DNA can result in substantial noise, producing both false positives and false negatives, even after steps are taken to decrease noise. The last key deficiency of a DEL screening is the need to re-synthesize hits without their DNA tag to confirm binding, which can be laborious and costly.

To circumnavigate some of these limitations, the conventional DEL approach can be complemented by machine learning, known as DEL-ML^18^. The key components of the DEL-ML approach include (i) training quantitative structure-activity relationship (QSAR) models on DEL screening data; (ii) using these models to computationally screen commercially available compounds or design de novo molecules with desired properties and high affinity for the protein target. A consensus model incorporating QSAR models built on DEL screening data and structure-based approaches such as molecular docking (when a 3D structure of the target is available), can virtually screen billions of commercially available molecules (such as the Enamine REAL space^19^ currently comprising 46 billion compounds) to predict high affinity hits for a specific target of interest.

The typical workflow of a DEL screen consists of incubation of pools of billions of molecules with DNA tags from multiple chemical libraries with a protein of interest, isolation of the protein with bound molecules, washing away less avidly bound or non-specifically bound molecules, and then releasing the bound molecules so they can be subjected to another cycle of enrichment. The feasibility of this cycle depends on the target protein of interest. Indeed, poorly soluble proteins such as G-coupled protein receptors may be more challenging. Emerging approaches including stabilization of a protein in detergent^20^ or nanodisk^21^, or screening in live cells^17,22^, enable DEL screening for such problematic targets.

After multiple screening cycles, the DNA tags of the selected compounds are amplified and sequenced. Comparing the sequence count of the DNA corresponding to selected compounds (or their respective building blocks/synthons) with a control experiment conducted in the absence of protein produces an enrichment score for each DNA/molecule. The data management workflow includes controlled vocabulary such as the source library of pooled compounds, the selection status of compounds or the enrichment status of synthons (yes/no). Records include the ID and chemical structures of the compounds, the sequence count of selected DNA tags or the enrichment score of selected hit molecules or synthons (Fig. 1).

Before any DEL screening data are ingested, a cloud-based database of the fully enumerated DEL compounds, with the DNA tag linker replaced with a methyl cap, should be assembled to avoid costly on-the-fly enumeration. Each library can be saved as an individual table with a column for the chemical structure in the SMILES format and a unique standardized ID. The chemical structure should be standardized following best practices (e.g., structure validation, ring aromatization and normalization of specific chemotypes)^23^, to allow downstream cheminformatics tasks including calculation of chemical descriptors and the development of machine learning models. The IDs should be unique across all libraries in the database for effective indexing and rapid queries. Ideally, they should also reflect the source library and the DEL building blocks that make up the individual DEL compound. Additional columns including building block ID’s, linker information, DNA tag information, or other compound information can also be included. A table used to map building block ID’s to chemical structures in a many-to-one relation can be used to minimize storage redundancy. In many cases, numerical embeddings of DEL compounds are required for example to conduct chemical similarity searching on the database. Additional tables mapping DEL ID’s to compressed representations of precomputed chemical fingerprints, like extended connectivity fingerprints (ECFP), can be helpful. This table would come at the expense of additional storage but may be a wise investment when the on-the-fly compute cost of fingerprints is high. DEL libraries can easily encode billions of chemicals and, depending on the additional information included, can easily span tens of terabytes even when compressed. The use of a cloud-based data warehouse (like BigQuery or RedShift) is crucial for the efficient scaling of both compute and storage resources.

The readout of DEL selections is a large collection of sequence reads. These reads are converted into enrichment values for each DNA tag, a proxy for the binding affinity of DEL compounds, using a variety of approaches. The data at this stage contains a lot of noise. Commonly, a negative control with no protein is used to remove some of the background noise, and compounds that are enriched across screens of diverse proteins are also flagged and removed. The thousands of compounds enriched in the presence of protein are then inspected for the shared presence of identical or highly similar building blocks. If no sensible structure-activity relationship can be found and there are relatively few enriched compounds, the DEL data is not likely to provide actionable information. If the selection appears successful, the enriched compounds can be extracted, along with a set of non-enriched compounds to generate a classification set of “active” and “negative” compounds, respectively.

## Best practices and recommendations for data archiving and dissemination

**Fig. 2:**
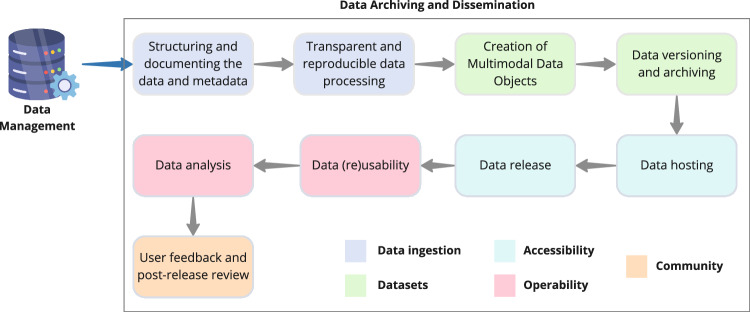
Workflow for data archiving and dissemination. The data archiving and dissemination workflow is a multistep process including data ingestion, creation of well-documented datasets that are made accessible and interoperable for the scientific community to use efficiently.

To best ensure the perennity of the rich data and metadata generated during protein production, hit discovery and hit optimization, one has to carefully plan their archiving and, in the context of open science research activities, maximise their dissemination and impact. While embracing the Open Science^24^ and FAIR^3^ principles, we here delve into best practices and recommendations for data archiving and dissemination (Fig. 2).

### Structuring and documenting the data and metadata

While ontologies are adopted during data generation and management (Fig. 1), the choice of standards and dictionaries must be well documented. These not only ensure consistency but also facilitate data sharing, interpretation, and integration across different platforms and projects.

### Transparent and reproducible data processing

Aggregating and harmonising large datasets to make them amenable to downstream analysis is a complex, lengthy and error prone process^25^. It is well established that scientists spend a large proportion of their time transforming and structuring data from one raw format into a desired format with the intent of improving data quality and making it more consumable (“data wrangling”)^26^. To fully realise the value of datasets, the code used to perform quality controls (QC) normalisation and harmonisation of the data should be made publicly available (for example via a code repository such as GitHub), clearly documented and associated with each dataset. This enables users to scrutinise the way data are processed and annotated and report potential errors or biases that inevitably creep in large-scale datasets.

### Creation of multimodal data objects

Considering the complexity of hit discovery and optimization, the data generated for each protein is intrinsically multimodal. The use of diverse high-throughput screening assays such as affinity selection mass spectrometry (ASMS) and DEL for hit discovery is just an example of multimodality. Integrating these diverse datasets can create comprehensive “protein data packages”. Having multiple data types included in a single data object allows for verification of its integrity and completeness. BioCompute objects^27,28^ allow for detailed tracking of processing pipelines used to generate datasets, which could be adopted for the low- and high-throughput data generated by the SGC and other Target 2035 projects.

### Data versioning and archiving

Datasets are not static and can evolve over time. Data updates include but are not limited to, new quality controls or normalisation approaches that impact past and future data collections, improvement of ontologies, and the use of next-generation profiling technologies. All these factors contribute to the production of multiple versions of a given dataset. While it is beneficial to produce datasets that increase in quality and size, it becomes challenging to ensure full data provenance, a key aspect of research transparency and reproducibility. It is therefore recommended to set up a versioning system that automatically tracks and reports on all the changes in the datasets for each release (change logs). Data nutrition labels^29^, which are simple standard labels that allow for visualisation and summarisation of key characteristics and updates of datasets, could be adopted for low- and high-throughput data generated by the SGC. Archiving of all versions of the data, as well as the change logs and data nutrition labels, is crucial to ensure that data analyses can be made fully reproducible, thereby better supporting scientific investigations over a long period.

### Data hosting

With the creation of the first protein sequence database in 1971, namely the Protein Information Resource^30^, the fields of genomics and chemical biology were at the forefront of the bioinformatics revolution. Based on this seminal work, well-established repositories have been implemented for specific data types, such as the Protein Data Bank (PDB)^31^, PubChem^32^ and ChEMBL^5^. The PDB is a repository that stores three-dimensional structural data of biological molecules, primarily proteins and nucleic acids, providing a resource for researchers to access and analyse large amounts of structural data generated from X-ray crystallography, nuclear magnetic resonance (NMR) spectroscopy, and cryo-electron microscopy. PubChem, developed by the US National Centre for Biotechnology Information (NCBI), and ChEMBL, developed by the European Bioinformatics Institute (EBI), are both open-access databases compiling detailed data and metadata on chemical compounds. They include details on molecular structures, properties, biological activities, and results from bioassays. They differ mainly on their scale and level of curation, PubChem provides a more comprehensive collection of data while ChEMBL focuses on a smaller set of human-curated datasets. Researchers can access these resources to analyse chemical data, predict biological interactions, and explore structure-activity relationships.

When a dataset is ready for release, it can be hosted in the relevant repository. However, these existing repositories do not necessarily handle the storage, access and mining of highly customised data types such as integrated data objects. We therefore envision the development of a centralised database to store and mine the protein data packages and act as a primary source to feed well-established repositories. This database will handle the structuring and documentation of metadata, transparent and reproducible data processing, the creation of multimodal data objects and their versioning, and the transformation of datasets to feed the existing repositories. This hybrid system will ensure data integration and verification in a centralised database and dissemination of specific data types via long-standing resources to increase the resilience and visibility of the data.

### Data release

Timely data release is crucial. Immediate releases upon generation and QC, akin the NIH policies^33^, promote transparency and community engagement but are logistically more complex as numerous versions of the data are produced. Another approach consists of releasing data with a pre-specified frequency (e.g., every quarter) or aligning releases with flagship publications that can offer context. Organization of open benchmarking challenges, such as CACHE^34^, also provides an opportunity to release datasets used to train and evaluate machine learning and artificial intelligence models.

### Data (re)usability

For data to be useful, users must be able to analyse them as efficiently as possible. Providing context for each dataset can be achieved through documentation (e.g., experimental protocols, laboratory notebooks) and publications. In addition, code to analyse the data and associated output files from tutorials and workshops provides excellent education materials for users. This ensemble of educational and training material derived from datasets will allow users to understand the data and the analyses that have already been performed by the scientific community^35^.

### Data analysis

To facilitate the analysis of data generated by the SGC and other Target 2035 linked efforts, one could use cloud services leveraging a centralized database to support multiple analytical protocols. The more traditional “Data2Model” way of working with data consists of downloading the data from a repository and analysing them on local infrastructure, such as on-prem high-performance computing^36^. As the data becomes increasingly large, reaching multiple terabytes, it is cumbersome to move the data where the analysis is done and best to bring the analysis code where the data is hosted (Model2Data)^36^. This is made possible by the adoption of scalable cloud platforms, such as Google Cloud, Amazon Web Services, and Microsoft Azure, where data processing resources can be attached to the data-storage buckets for intensive analysis without moving the data.

### User feedback and post-release review

Continuous improvement is crucial to ensure the highest data quality and useability. Actively seeking user feedback and encouraging post-publication reviews can reveal opportunities for improvement and potential biases and limitations of the datasets. The future SGC and Target 2035 platforms will include a process to collect general feedback as well as dataset-specific feedback via a ticketing system.

As the bedrock of drug development, data demands meticulous management and dissemination. Adhering to best practices ensures that the wealth of data generated not only guide scientific research and development to success but also serves as the substrate for future AI-driven technological breakthroughs. This is a complex activity that not only requires advanced expertise and resources but also an early and substantial investment.

## Opportunities and challenges in data science

### Consistent data processing: a critical prelude to building AI models

The critical nature of precise storage, management, and dissemination of data in the realm of drug discovery is universally recognized. This is because the extraction of meaningful insights depends on the data being readily accessible, standardized, and maintained with the highest possible consistency. However, the implementation of good data practices can vary greatly and depends on the goals, culture, resources, and expertise of research organizations. A critical, yet sometimes underestimated, aspect is the initial engineering task of data preprocessing, which entails transforming raw assay data into a format suitable for downstream analysis. For instance, quantifying sequencing reads from DNA-encoded library screens into counts is required for the subsequent hit identification data science analysis step. Ensuring the correctness of this initial data processing step is imperative, but it may be given too little priority, potentially leading to inaccuracies in subsequent analyses. Standardization of raw data processing is an important step to enable subsequent machine learning studies of DEL data. Currently, this step is done by companies or organizations that generate and screen DEL libraries, and the respective protocols are reported if a study is published (see the ”Methods” section in McCloskey et al. ^18^). Making data processing pipelines open source will help establish best practices to allow for scrutiny and revisions if necessary. While this foundational step is vital for harnessing data science, it is worth noting that it will not be the focus of this discussion.

### Examples of AI application in early-stage drug discovery

In drug discovery, data science presents numerous opportunities to increase the efficiency and speed of the discovery process. Initially, data science facilitates the analysis of huge experimental data, e.g., allowing researchers to identify potential bioactive compounds in large screening data. Machine learning models can be trained on data from DEL or ASMS and, in turn, be used for hit expansion in extensive virtual screens. For example, a model trained to predict the read counts of a specific DEL screen can be used to identify molecules from other large compound libraries, which are likely to bind to the target protein under consideration^18^.

As the drug discovery process advances to compound optimization, data science can be used to analyse and predict the pharmacokinetic and dynamic properties of potential drug candidates. This includes model-based evaluation of absorption, distribution, metabolism, excretion, and toxicity (ADMET) profiles. ADMET parameters are crucial in prioritizing and optimizing candidate molecules. Acknowledging their importance, the pharmaceutical industry has invested substantially in developing innovative assays and expanding testing capacities. Such initiatives have enabled the characterization of thousands of compounds through high-quality in-vitro ADMET assays, serving as a prime example of data curation in many pharmaceutical companies^37^. The knowledge derived from accumulated datasets has the potential to impact research beyond the projects where the data was originally produced. Computational teams utilize these data to understand the principles governing ADMET endpoints as well as to develop in-silico models for the prediction of ADMET properties. These models can help prioritize compound sets lacking undesired liabilities and thus guide researchers in their pursuit to identify the most promising novel drug candidates.

### Combining correlated data types to improve AI models

Major approaches in early drug discovery data science encompass classification, regression, or ranking models. They are, for example, employed in drug discovery to classify molecules as mutagenic, predict continuous outcomes such as the binding affinity to a target, and rank compounds in terms of their solubility. Incorporating prior domain knowledge can further enhance the predictive power of these models. Often, assays or endpoints that are correlated can be modelled together, even if they represent independent tasks. By doing so, the models can borrow statistical strength from each individual task, thereby improving overall performance compared to modelling them independently. For example, multitask learning models can predict multiple properties concurrently, as demonstrated by a multitask graph convolutional approach used for predicting physicochemical ADMET endpoints^38^.

### Choosing the most relevant data representation

When confronted with training data that have ambiguous labels, utilizing multiple-instance learning can be beneficial. Specifically, in the context of bioactivity models, this becomes relevant when multiple 3D conformations are considered, as the bioactive conformation is often unknown^39^. A prevalent challenge in applying data science for predictive modelling of chemical substances is choosing a suitable molecular representation. Different representations, such as Continuous Data-Driven Descriptor (CDDD)^40^ from SMILES strings, molecular fingerprints^41^ or 3D representations^42^, capture different facets of the molecular structure and properties^43^. It is vital to select an appropriate molecular representation as this determines how effectively the nuances of the chemical structures are captured. The choice of the molecular representation influences the prediction performance of various downstream tasks, making it a critical factor in AI-driven drug discovery, as discussed in detail in David et al.’s^43^ review and practical guide on molecular representations in AI-driven drug discovery. Recent studies have found that simple k-nearest neighbours on molecular fingerprints can match or outperform much more complicated deep learning approaches on some compound potency prediction benchmarks^44,45^. On the other hand, McCloskey et al. ^18^ have discovered hits by training graph neural networks on data from DEL screens, which are not close to the training set using established molecular similarity calculations. Whether a simple molecular representation, infused with chemical knowledge, or a complex, data-driven deep learning representation is more suitable for the task at hand depends strongly on the training data and needs to be carefully evaluated on a case-by-case basis to obtain a fast and accurate model.

### Defining the right training and test sets

Sound strategies for splitting data into training and test sets are crucial to ensure robust model performance. These strategies include random splitting, which involves dividing the data into training and test sets at random, ensuring a diverse mix of data points in both sets. Temporal splitting arranges data chronologically, training the model on older data and testing it on more recent data, which is useful for predicting future trends. Compound cluster-wise splitting devides training and test sets into distinct chemical spaces. Employing these strategies is essential as inconsistencies between the distributions of training and test data can lead to unreliable model outputs, negatively impacting decision-making processes in drug discovery^46^.

### Estimating prediction uncertainty

The successful application of machine learning requires keeping their domain of applicability in mind at all stages. This includes using the techniques described in the previous section for data curation and model development. However, it is equally important to be able to estimate the reliability of a prediction made by an AI model. While generalization to unseen data is theoretically well understood for classic machine learning techniques, it is still an active area of research for deep learning. Neural networks can learn complex data representations through successive nonlinear transformations of the input. As a downside of this flexibility, these models are more sensitive to so-called adversarial examples, i.e., instances outside the domain of applicability that are seemingly close to the training data from the human perspective^44^. For this reason, deep learning models often fall short of providing reliable confidence estimates for their predictions. Several empirical techniques can be used to obtain uncertainty estimates: Neural network classifiers present a probability distribution indicative of prediction confidence, which is inadequately calibrated but can be adjusted on separate calibration data^45^. For regression tasks, techniques such as mixture density networks^47^ or Bayesian dropout^48^ can be employed to predict distributions instead of single-point estimates. For both classification and regression, the increased variance of a model ensemble indicates that the domain of applicability has been left^49^.

### Designing virtuous DMTA cycles

With the methods described in the previous paragraphs, we possess the necessary methodological stack to establish a data-driven feedback loop from experimental data, a crucial component for implementing active learning at scale. By leveraging predictive models that provide uncertainty estimates, we can create a dynamic and iterative data science process for the design-make-test-analyse (DMTA) cycle. For instance, these predictive models can be utilized to improve the potency of a compound by identifying and prioritizing molecules that are predicted to have high affinity yet are uncertain. Similarly, the models can be used to increase the solubility of a compound by selecting molecules that are likely to be more soluble, thus improving delivery and absorption. This process continuously refines predictions and prioritizes the most informative data points for subsequent experimental testing and retraining the predictive model, thereby enhancing the efficiency and effectiveness of drug discovery efforts. An important additional component is the strategy to pick molecules for subsequent experiments. By intelligently selecting the most informative samples, possibly those that the model is most uncertain about, the picking strategy ensures that each iteration contributes maximally to refining the model and improving predictions. For example, in the context of improving compound potency, the model might prioritize molecules that are predicted to have high potency but with a high degree of uncertainty. These strategies optimize the DMTA process by ensuring that each experimental cycle contributes to the refinement of the predictive model and the overall efficiency of the drug discovery process.

### Scalability of AI predictions for virtual screening

**Fig. 3:**
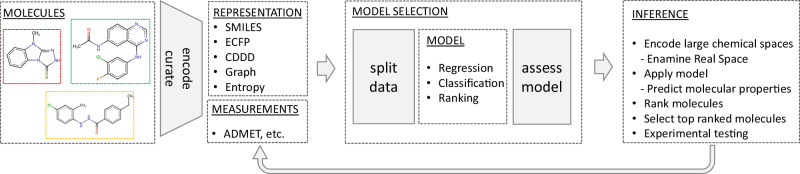
Workflow for computational molecular property prediction. Computational workflow for predicting molecular properties, starting with molecular structure encoding, followed by model selection and assessment, and concluding with the application of models to virtually screen libraries and rank these molecules for potential experimental validation. The process can be cyclical, allowing iterative refinement of models based on empirical data. ADMET: absorption, distribution, metabolism, and excretion–toxicity. ECFP: Extended Connectivity Fingerprints. CDDD: Continuous Data-Driven Descriptor, a type of molecular representation derived from SMILES strings. Entropy: Shannon entropy descriptors^50,51^.

When applying the computational workflow depicted in Fig. 3 on large compound libraries, scientists encounter a rather uncommon scenario for machine learning: usually, the training of deep neural networks incurs the highest computational cost since many iterations over large datasets are required, while comparatively few predictions will later be required from the trained model within a similar time frame. However, when inference is to be performed on a vast chemical space, we face the inverse situation. Assessing billions of molecules for their physicochemical parameters and bioactivity is an extremely costly procedure, potentially requiring thousands of graphics processing unit (GPU) hours. Therefore, not only predictive accuracy but also the computational cost of machine learning methods is an important aspect that should be considered when evaluating the practicality of a model.

## Conclusion

While the roadmap presented here is primarily designed to guide data management, data dissemination, and data science operations at the Structural Genomics Consortium, a pre-competitive public-private partnership focused on open-science drug discovery, it is drawn from the collective experience of scientists across multiple sectors and can be applied, fully or partially, to research operations in diverse settings. Domain experts may be more inclined to embrace data science if ML models are able to predict seemingly erratic behaviours such as activity cliffs in chemical series and if predictions are not perceived as arbitrary but grounded on an explainable interpretation of the physics and chemistry underlying experimental results. Indeed, the domain knowledge of organic, process and medicinal chemists could be best leveraged by explainable AI. Beyond the adoption of best practices presented here, we believe that future breakthroughs will depend on the adoption of a different mindset, where experimentalists see for themselves how data science augments the impact of their work, where data scientists understand the language of experimentalists, and where data science applications are incorporated into the experimental design.
